# Pharmacological Foundation and Novel Insights of Resveratrol in Cardiovascular System: A Review

**DOI:** 10.2174/011573403X343252250502045328

**Published:** 2025-05-14

**Authors:** Ruchi Tiwari, Gaurav Tiwari, Anju Singh, Namdev Dhas

**Affiliations:** 1 Department of Pharmacy, PSIT-Pranveer Singh Institute of Technology, NH 19, Kanpur, 209305, Bhauti, Uttar Pradesh, India;; 2 University Institute of Pharmacy, Chhatrapati Shahu Ji Maharaj University (Formerly Kanpur University), Kanpur, 208024, Uttar Pradesh, India;; 3 Department of Pharmaceutics, Manipal College of Pharmaceutical Sciences, Manipal Academy of Higher Education (MAHE), Manipal, Udupi, 576104, Karnataka State, India

**Keywords:** Resveratrol, atherosclerosis, hypertension, heart failure, lipoproteins, cholesterol, calorie restriction, inflammation, cardioprotection

## Abstract

Research into drugs that can enhance cardiovascular health has been sparked by the rising prevalence of cardiovascular illnesses (CVDs). In addition to its anti-inflammatory and antioxidant qualities, Resveratrol (RES) is well known for its capacity to increase endothelial NO synthase (eNOS) activity. This page summarises RES's wide effects on energy metabolism, resilience to stress, exercise mimicking, circadian rhythm, lifespan control, and microbiome composition. This article addresses the poor and contradictory results shown in preclinical and clinical trials provides an update on the cardiovascular preventive properties of RES. The activation of AMP-activated protein kinase (AMPK), silent information regulator 1 (SIRT1), and natural antioxidant enzymes is associated with some of the positive effects of RES on the cardiovascular system. A microarray data summary indicates a strong correlation between the heart's reaction to calorie restriction and the transcriptional responses to RES. RES has been demonstrated to reduce contractile dysfunction, cardiac remodelling, and hypertrophy in several animal models of heart failure. Its preventive properties are believed to be due to several molecular pathways, including the suppression of prohypertrophic signalling molecules, enhancement of cardiac Ca^2+^ handling, control of autophagy, and decreases in inflammation. RES thus has the potential to be used in several novel therapeutic approaches for treating diseases such as atherosclerosis, ischemia/reperfusion damage, metabolic syndrome, heart failure, and inflammatory changes associated with ageing.

## 1 INTRODUCTION

**Fig. (1):**
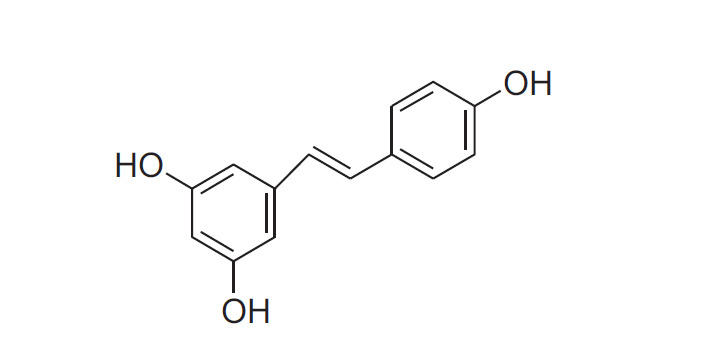
Chemical structure of resveratrol.

RES is a polyphenolic substance that isn't a flavonoid and is derived from stilbenes. It's a phytoalexin that plants make and is mostly found in red wine and grapes [1, 2]. Red wine has modest quantities of RES, but using high-dose supplements, which are frequently employed for research studies, might make it difficult to interpret the data [3]. Furthermore, very little published research on RES is connected to human clinical trials, despite an exponential growth in these investigations [4, 5]. Therefore, long-term, carefully monitored studies are required to validate its favourable effect on CVDs. RES, also known as trans-3, 4', 5-trihydroxystilbene, is a naturally occurring phytoalexin that is a hydroxylated derivative of stilbene (Fig. **1**) [6].

Given that RES has long been recognized as an anti-ageing substance, its cardioprotective benefits may be explained by its related anti-inflammatory, antioxidant, anti-proliferative, angioregulatory, and autophagy-enhancing characteristics [7]. More significantly, additional research showed that RES therapy is linked to decreased systemic circulation diastolic blood pressure and decreased expression of inflammatory markers in endothelial cells [6, 8], including intercellular adhesion molecule (ICAM) and IL-8, which accounts for decreased risks of ischemic stroke and hypertension [9-11]. Apart from the SIRT1 pathway, RES also controls signaling in the cardiovascular system that involves 5' adenosine monophosphate-activated protein kinase (AMPK) and peroxisome proliferator-activated receptor (PPAR-γ) [12, 13]. Additionally, there is proof that RES can alter the biological circadian rhythm [14], which is a key theory that could influence the onset of CVDs. CVD is an umbrella term encompassing conditions such as deep vein thrombosis, peripheral artery disease, coronary artery disease (CAD), congenital heart disease, and cerebrovascular disease [15]. A class of illnesses known as CVDs affects the heart and/or blood vessels [16, 17].

It is anticipated that the prevalence of CVD would rise further, leading to an expected 23.3 million CVD-related deaths globally by 2030 due to rising CVD incidence. These figures make it clear that novel treatments are required to counteract the significant impact that CVDs have on people's quality and longevity worldwide [18]. RES and other polyphenolic chemicals found in red wine have attracted a lot of interest, particularly because of their possible function in protecting against cardiovascular disease [19, 20]. Nevertheless, red wine contains relatively little RES [21], indicating that dietary and lifestyle variables other than RES alone may mediate the French Paradox. As a matter of fact, RES is found in meals and drinks, but in very small amounts, and most studies on the substance have focused on RES supplements [22-24]. In a similar vein, several molecular targets for RES have been found through mechanistic investigations [25]. However, there has been very little evidence to support these encouraging effects in human clinical studies, with a few negative outcomes and several conflicting findings [26]. The review will go over key molecular targets of RES that are connected to its preventive effects against cardiovascular disease. In the United States, there are currently 150 million overweight people, 18 million of whom have been diagnosed with diabetes, and an additional 7 million are expected to have the disease undetected, making up around 11% of the population [27]. Clinical evidence indicates that obesity and diabetes are significant risk factors for cardiovascular complications and mortality. They are strongly associated with conditions such as myocardial infarction, congestive heart failure, left ventricular hypertrophy (LVH), and renal failure. Given these risks, addressing these conditions is of critical importance [28]. This is a forty percent rise over the present. Therefore, the enormous potential benefit of novel medicines that might cure metabolic diseases and simultaneously lessen cardiovascular system damage is underscored by the human and related economic consequences of CVD. Benefits of calorie restriction on the heart. The most effective and repeatable strategy shown to increase longevity and postpone the detrimental physiological effects of ageing-related chronic illnesses is calorie restriction (CR) [29]. Given that CR includes several interrelated and mutually dependent signalling, metabolic, physiologic, genetic, and cellular pathways, it is challenging to precisely identify the mechanism or mechanisms generating CR [30, 31]. Heart failure (HF) is a condition that is becoming more and more common in Western countries, both medically and socially, despite great advancements in therapy [32]. The adrenergic system and RAAS were pharmacologically inhibited, which significantly decreased mortality [33]. Prolonged exposure to reactive oxygen species (ROS) can lead to significant oxidative damage to proteins, DNA, and lipids, which in turn impacts cardiomyocyte contractility, ion transport, and calcium cycling [34]. Additionally, ROS activate various detrimental intracellular signalling pathways that contribute to apoptosis and necrosis. In heart failure (HF), modulating stress-responsive signalling pathways and regulating intracellular ROS production may help mitigate or prevent these harmful effects [35]. Various experimental studies have demonstrated that RES influences multiple pathological mechanisms involved in cardiovascular conditions, including myocardial ischemia, myocarditis, cardiac hypertrophy, and heart failure [36-41].

The benefits of RES for HF protection have been attributed to various mechanisms, such as lowering apoptosis, decreasing oxidative stress and inflammation, blocking pathological hypertrophic signalling, increasing Ca^2+^ handling, modifying autophagy through various intracellular pathways, and inhibiting pathological hypertrophic signalling [39-43]. The goal of the current work was to examine RES in an animal model of postinfarction heart failure where patchy and fibrosis were caused by myocardial infarction produced by the powerful sympathetic medication isoproterenol [44]. New preventative or therapeutic strategies are still needed in both industrialized and developing nations, despite a plethora of scientific and medical advancements targeted at mitigating the negative consequences of CVDs. Specifically, in other species, large dosages of RES have been linked to longer lifespans.

### 1.1 Impact of RES on the Cardiovascular System

#### 1.1.1 Anti-atherosclerotic Effects

**Fig. (2):**
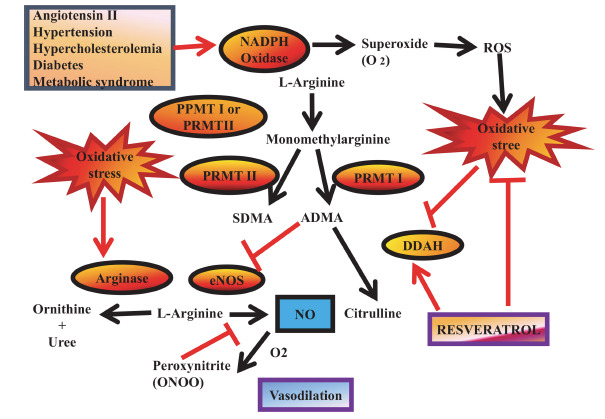
Resveratrol (RES) has potential benefits as an antioxidant and a modulator of the dimethylaminohydrolase (DDAH)/asymmetric dimethylarginine (ADMA) pathway. Oxidative stress negatively impacts nitric oxide (NO) bioavailability, which is crucial for vascular health. Cardiovascular diseases (CVDs) can lead to increased ADMA levels due to DDAH inhibition, triggering NADPH oxidase activity and contributing to oxidative stress. This process can cause endothelial nitric oxide synthase (eNOS) uncoupling, reducing NO production, a consequence linked to both ADMA and peroxynitrite accumulation. RES may help counteract these effects by enhancing DDAH activity and functioning as an antioxidant through radical scavenging and activation of antioxidant defence mechanisms. Key components involved in this pathway include ADMA, symmetric dimethylarginine (SDMA), eNOS, protein methyltransferase (PRMT), reactive oxygen species (ROS), and DDAH.

Extracellular lipid buildup, smooth muscle cell migration and proliferation, and chronic inflammation are the primary hallmarks of atherosclerosis. It is essential to maintain a healthy lipid profile because lipids, especially low-density lipoproteins (LDLs), are a major contributor to atherosclerosis [28]. Preclinical research suggests that RES may help regulate lipid levels by increasing HDL cholesterol while reducing plasma triglycerides and LDL cholesterol. RES appears to inhibit 3-hydroxy-3-methyl-glutaryl-CoA reductase (HMG-CoA reductase), an enzyme involved in the early stages of cholesterol synthesis, potentially enhancing the cholesterol-lowering effects of pravastatin. Furthermore, *in vitro* studies indicate that RES can upregulate LDL receptor (LDL-R) expression in liver cells, which may contribute to lowering LDL cholesterol levels in the bloodstream [32]. Beyond lipid regulation, RES exhibits antioxidant activity that reduces LDL oxidation, a key process in the development of atherosclerosis, while also enhancing the body's natural antioxidant defences. Additionally, its anti-inflammatory effects and ability to inhibit smooth muscle cell migration further support its potential as an anti-atherogenic agent (Fig. **2**). RES influences cardiovascular health by modulating multiple pathways associated with atherosclerosis. These include the activation of sirtuin 1 (SIRT-1), endothelial nitric oxide synthase (eNOS), nuclear factor erythroid 2 2-related factor 2 (Nrf2), and the antioxidant response element (ARE), while also reducing the production of tumor necrosis factor-alpha (TNFα). These mechanisms contribute to protecting endothelial cells from apoptosis, reducing vascular inflammation, and improving overall endothelial function [43-45]. It has also been demonstrated that RES works in the early stages of atherosclerosis by blocking the NF-κB signaling pathway, which lowers the expression of adhesion molecules like intercellular adhesion molecule-1 (ICAM-1) and vascular cell adhesion molecule-1 (VCAM-1). Moreover, RES influences inflammatory processes by regulating microRNA (miRNA) expression, such as miR-663, which plays a role in inflammation. Its anti-inflammatory properties are also linked to Nrf2 activation, which helps regulate cytokine production in cardiomyocytes, potentially protecting against sepsis-induced cardiomyopathy and reducing cardiac damage caused by endotoxins. By limiting vascular smooth muscle cell migration and proliferation, RES further strengthens its anti-atherogenic role [46]. Despite these promising findings, a meta-analysis of seven clinical trials reported mixed results, with some studies showing no significant effects, while others indicated improvements in lipid profiles. For instance, daily RES supplementation (250–1000 mg) was associated with reduced LDL cholesterol levels in some individuals. Additional trials have found that RES (at doses of 150 mg/day in obese men and 500 mg/day in smokers) effectively lowers plasma triglycerides [47]. Moreover, a study on patients undergoing statin therapy for primary prevention found that supplementation with 350 mg/day of a resveratrol-enriched grape extract (providing 8 mg of resveratrol) led to a 20% reduction in oxidized LDL and a 4.5% decrease in LDL cholesterol. However, further clinical research is necessary to confirm RES's overall anti-atherosclerotic benefits, as its effects extend beyond lipid regulation [48].

#### 1.1.2 Anti-hypertensive Effects

**Fig. (3):**
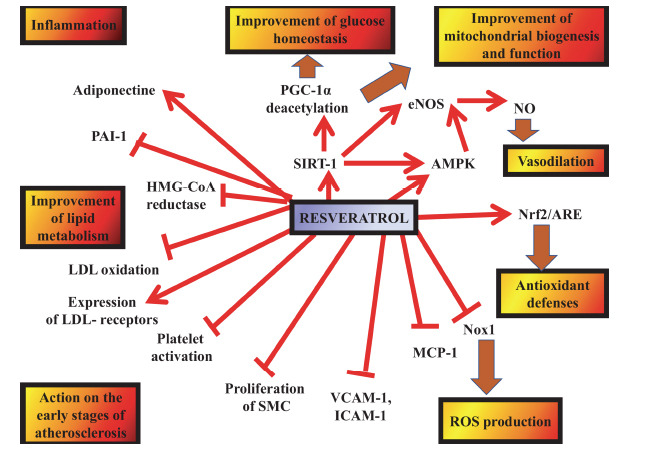
The reticuloendothelial system (RES) may influence metabolic dysfunction and contribute to the development of atherosclerosis. Key biological molecules involved in these processes include intercellular adhesion molecule-1 (ICAM-1), low-density lipoprotein (LDL), 3-hydroxy-3-methyl-glutaryl-CoA (HMG-CoA) reductase, endothelial nitric oxide synthase (eNOS), and AMP-activated protein kinase (AMPK). Additionally, monocyte chemotactic protein-1 (MCP-1) and nuclear factor kappa B (NF-κB) play roles in inflammatory responses, while smooth muscle cells (SMCs), reactive oxygen species (ROS), and plasminogen activator inhibitor-1 (PAI-1) contribute to vascular remodelling and thrombosis. Other key regulators include peroxisome proliferator-activated receptor gamma (PPAR-γ) and its co-activator PGC-1α, as well as sirtuin 1 (SIRT-1), which functions as a vasoconstrictor. Vascular cell adhesion molecule-1 (VCAM-1), nuclear factor erythroid 2-related factor 2 (Nrf2), and NADPH oxidase 1 (Nox1) are also crucial in oxidative stress and endothelial function.

One of the main risk factors for CVDs is hypertension [46]. A few investigations have demonstrated that RES can correct the anatomical and functional defects linked to hypertension, namely cardiac hypertrophy and contractile dysfunction [47-50]. In a recent study involving 28-week-old spontaneously hypertensive rats, RES alone did not significantly reduce blood pressure [51]. However, when combined with the blood pressure-lowering medication hydralazine, RES was more effective in improving cardiovascular parameters than hydralazine alone. It is noteworthy that in all of these investigations, the length of the RES therapy was rather brief. The energy metabolism regulator AMPK, SIRT-1, Nrf2, and other endothelium-dependent pathways may be implicated in the antihypertensive effects of RES (Fig. **3**) [52]. This leads to a vasodilation through enhanced NO availability in correlation with elevated eNOS expression and activity [7, 8], and this characteristic is linked to RES's antioxidant capabilities [52]. SIRT-1 activation mediated by RES increased eNOS expression and activity [53]. Additionally, RES has the power to activate AMPK, which raises the generation of NO [54]. Researchers also demonstrated that inhibiting AMPK reversed the effects of RES in preventing AngII-induced aortic contractions. As a result, in a hypertensive mouse model induced by AngII, daily RES treatment effectively reduced hypertension [55, 56].

In clinical studies, a meta-analysis of six randomized controlled trials involving 247 participants found that while lower doses of RES had no significant effect, higher doses (approximately 150 mg/day) effectively reduced blood pressure [57]. One confounding factor is that blood pressure reduction is often accompanied by improvements in metabolic markers. Notably, in hypertensive and dyslipidemic subjects, the antihypertensive effect of RES, evidenced by increased acetylcholine-induced vasorelaxation, was more pronounced [58], which may be linked to earlier findings in animal models [47].

#### 1.1.3 Effect on Heart Failure

**Fig. (4):**
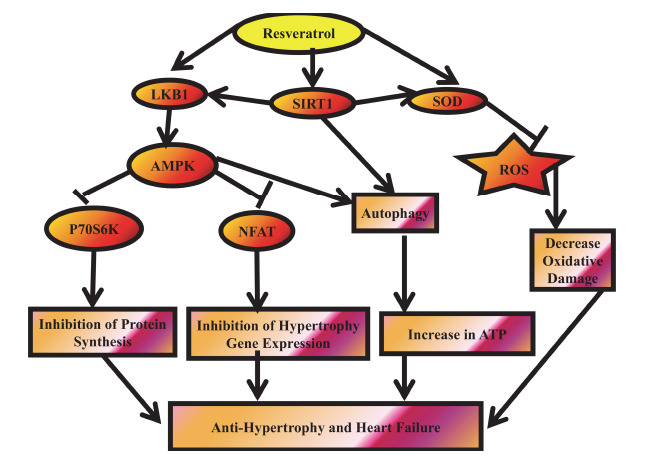
Proposed mechanisms of RES in preventing cardiac hypertrophy and heart failure. RES interferes with multiple pathways involved in the development of cardiac hypertrophy and heart failure. One key mechanism involves the activation of AMP-activated protein kinase (AMPK) *via* the LKB1 pathway. This activation suppresses hypertrophic gene expression and protein synthesis by inhibiting the nuclear factor of activated T cells (NFAT).

Heart failure (HF) can be summed up as the heart's incapacity to adequately pump blood to the body's other organs. Significantly, persons in North America over 45 have a 20% lifetime chance of getting HF [58, 59], and throughout the next one to two decades, this condition is predicted to increase [60]. Numerous heart-related therapies are available and have demonstrated efficacy in decreasing heart-related mortality [61]. Studies comparing RAAS blockade alone with dual inhibition of neprilysin and the renin-angiotensin-aldosterone system (RAAS) through angiotensin receptor-neprilysin inhibitors have shown a notable decrease in cardiovascular-related mortality, heart failure hospitalizations, and overall mortality. However, despite these advancements, the mortality rate following a heart failure (HF) diagnosis remains high. This challenge is further exacerbated by the limited efficacy of many pharmacological treatments for heart failure with reduced ejection fraction (HF-REF) in patients with heart failure with preserved ejection fraction (HF-PEF) [62]. Consequently, there is a pressing need for innovative therapies to enhance HF management and improve patients’ quality of life. RES supports autophagy *via* the AMPK and SIRT1 pathways while also mitigating oxidative stress by stimulating the antioxidant enzyme superoxide dismutase (SOD), thereby reducing reactive oxygen species (ROS) production (Fig. **4**) [63].

#### 1.1.4 Effects on Heart Failure and Left Ventricle Function

**Fig. (5):**
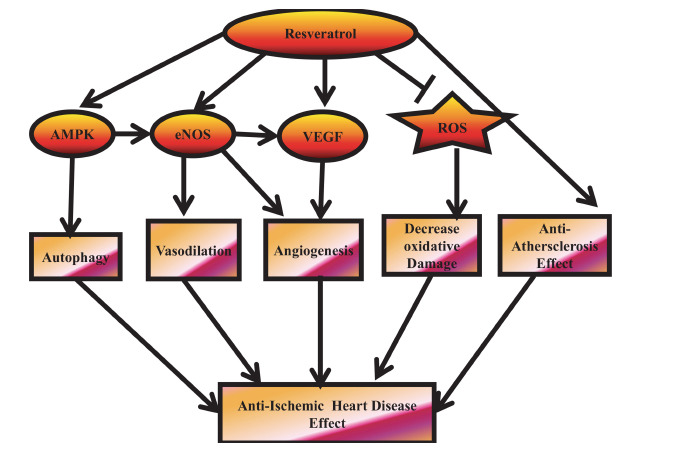
Supra-ischemic activities of RES. Endothelial NO synthase (eNOS), vascular endothelial growth factor (VEGF), and AMP-activated protein kinase (AMPK) are activated by RES to give its antiischemic activities, whereas reactive oxygen species (ROS) and atherosclerosis are inhibited.

These benefits include prolonged survival, enhanced diastolic and systolic function, minimized adverse atrial and left ventricular remodeling [64], improved hemodynamics and cardiac energetics, and greater exercise capacity [65-68]. However, a double-blind, placebo-controlled study found that a three-month regimen of 10 mg/day of RES significantly improved left ventricular diastolic function in individuals with stable coronary artery disease (Fig. **5**) [69]. Furthermore, individuals with angina pectoris showed a substantial reduction in b-type natriuretic peptide (BNP) after receiving 20 mg of RES per day for 60 days, indicating enhanced left ventricular function [70]. These findings, however small, raise the possibility that RES directly affects human cardiac function. This does not indicate that RES will help individuals with HF have better myocardial function, but it does offer intriguing information that points to the need for more clinical research in this field [71]. The RES-HF study aims to assess the safety and tolerability of long-term, high-dose RES use in individuals with heart failure. Additionally, it will provide valuable insights into the potential of RES as a supplementary treatment alongside standard heart failure therapy [72].

#### 1.1.5 RES on Lipoprotein and Cholesterol Presence

**Fig. (6):**
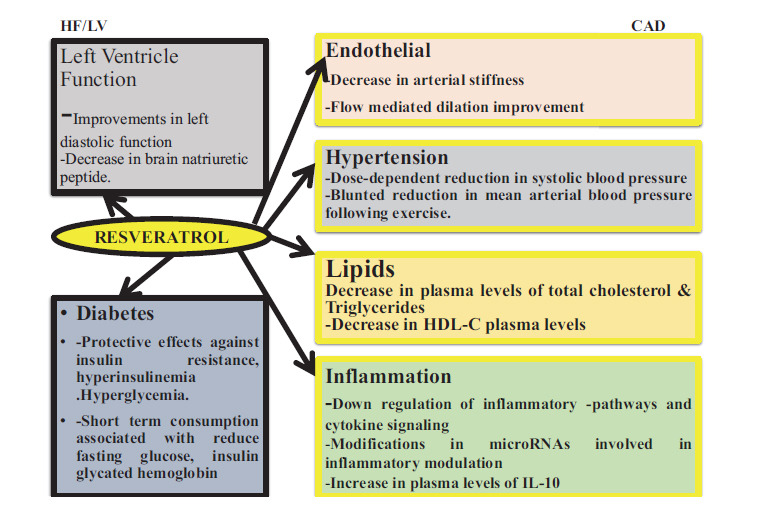
An overview of the conclusions derived from the RES clinical trials. The main conclusions from the research that used RES in various patient groups are outlined below. Diabetes, heart failure (HF)/left ventricular (LV) function, and coronary artery disease (CAD) are the three main groupings of illness states, and their impacts on many biological entities are noted.

Conditions like hypercholesterolemia are thought to have a significant influence in the pathophysiology of atherosclerosis [73, 74]. To explain, LDL that is exposed to an atherosclerotic lesion's macrophages undergoes oxidation (LDL-ox), which can harm endothelial cells and promote the development of atherosclerotic lesions [75]. Furthermore, increased lipoprotein retention in lesions caused by low-density lipoprotein (LDL) can lead to significant plaque accumulation and acute thrombotic vascular events, including myocardial infarction [76-80]. Research on randomised controlled trials (RCTs) assessing a drug's cardio-protective effects, including trials examining the effects of RES, frequently uses statin-therapy-like effects (Fig. **6**) [81]. The cardioprotective benefits of RES are frequently shown by elevated HDL levels since a decreased risk of coronary artery disease (CAD) is linked to a greater HDL-to-LDL ratio. Results from randomized controlled trials (RCTs) examining how RES supplementation affects lipid profile alterations seem to be inconsistent [82]. Nonetheless, RES has been linked to several possible pathways that may enhance human lipid profiles. These effects include an elevation in SIRT1 levels, which may facilitate reverse cholesterol transport [81, 83], an enhancement in lipid profile [84], and a reduction in the mRNA expression of hepatic 3-hydroxy-3-methyl-glutaryl-CoA (HMG-CoA) reductase [85], a key enzyme in cholesterol synthesis [86]. RES can mediate these possible routes; however, the clinical data is more in favour of no direct impact of RES on plasma levels of total cholesterol, triglycerides, LDL, and HDL than it is of a substantial effect. For instance, several RCTs have demonstrated that RES supplementation at different dosages does not affect the lipid profile [84, 85]. Remarkably, a reduction in HDL-C plasma concentrations was seen generally in this meta-analysis of RES RCTs [86, 87]. Additionally, research indicated that triglyceride levels increased following RES consumption, while a reduction in total cholesterol plasma levels was observed only in individuals with a healthy body mass index (BMI), with no significant effect in those who were overweight or obese [88]. Nevertheless, it should be mentioned that the triglyceride rise became negligible [89] when the research conducted by Singh and Lang [90] was eliminated from this meta-analysis. Furthermore, adverse effects like a slowed decline in plasma levels of total cholesterol and LDL-C after exercise [48, 91] and a rise in plasma levels of triglycerides and total cholesterol after exercise [65, 92] have been reported in several randomized controlled trials (RCTs) examining RES's impact on lipid profile. Considering the available data, particularly the meta-analyses, RES appears to have no effect, or maybe a negative one, on plasma lipid profile (assuming that plasma levels of total cholesterol, LDL-C, HDL-C, and triglycerides are utilized as indicators of lipid profile status [83]). This is noteworthy since the research also found that RES did not affect plasma levels of LDL-C, which is consistent with other clinical trials that show RES is ineffective at reducing the risk of lipoprotein-induced atherosclerosis [91, 93, 94]. Research demonstrated that RES may still lower the risk of atherosclerosis and other lipoprotein-related CVDs by reducing small LDL particles (LDL-P), which in turn lowers LDL-ox, even though prior studies did not find any decrease in LDL-C levels or other lipid profile changes after RES supplementation [92, 95]. To confirm these findings, further research is needed, as an additional randomized controlled trial (RCT) showed that RES supplementation did not impact lipid oxidation [93]. The data on RES's effects on lipids are frequently contradictory and inconsistent. Therefore, additional studies focusing on LDL oxidation and LDL particle sizes, alongside traditional lipid profiles, are necessary to determine whether RES can effectively reduce the risk of cardiovascular diseases associated with poor lipoprotein and cholesterol metabolism [96, 97].

#### 1.1.6 RES in Hypertension

**Fig. (7):**
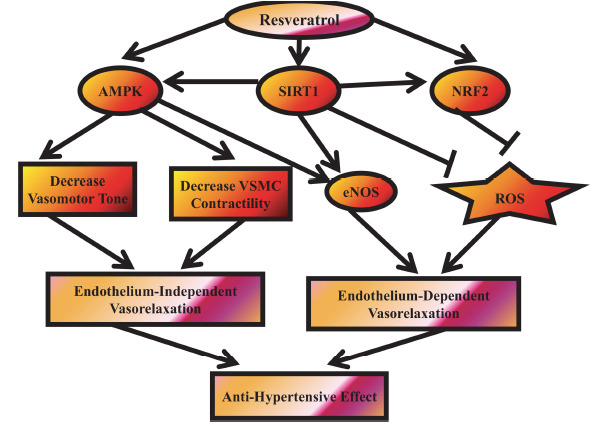
Potential mechanisms underlying the antihypertensive effects of RES involve its interaction with nuclear factor erythroid-2 related factor 2 (NRF2), AMP-activated protein kinase (AMPK), and silent information regulator 1 (SIRT1). These pathways contribute to vasorelaxation through both endothelium-dependent and endothelium-independent mechanisms, with notable crosstalk among them.

Research indicates that one of the main risk factors for CVDs is hypertension [94, 98]. In industrialized nations, the prevalence of hypertension is considered to be more than 25% of adults [95], while in the US, 33% of adults over 20 are expected to have hypertension [96]. Even after combining three or more anti-hypertensive medicines, some patients may not reach the target blood pressure (BP) levels, even though numerous pharmacological classes are utilized to treat hypertension [97, 99]. Furthermore, not all anti-hypertension drugs can shield against the end-organ damage that hypertensive patients frequently experience, such as hypertensive retinopathy, cardiac hypertrophy, hypertensive nephropathy, and cerebrovascular problems [97, 100]. Despite the significance of these results, hydralazine is not frequently utilized in clinical settings to treat hypertension. Remarkably, RES has demonstrated positive effects on the vasculature in numerous studies, even when it did not lower blood pressure (Fig. **7**). Comparable results have been observed in endothelium-intact aortic rings from healthy male and female rats, where RES supplementation (50 mg/L in drinking water) enhanced Ach-induced relaxation while reducing contractions caused by phenylephrine and Ang II [98, 99, 101]. In contrast, RES did not influence endothelium-dependent relaxation in thoracic aortic rings from normotensive Sprague Dawley rats, indicating that its effects may be more pronounced in compromised arteries than in healthy ones [100, 102].

A significant risk factor for CVDs has been demonstrated to be hypertension [101, 103]. In industrialized nations, the prevalence of hypertension is thought to be more than 25% of adults [102], while in the US, the prevalence is believed to be 33% of adults over 20 who have hypertension. Even after combining three or more anti-hypertensive medicines, some individuals may not reach the target blood pressure (BP) levels, although numerous pharmacological classes are used to treat hypertension [103, 104]. Furthermore, not all anti-hypertension drugs can shield against the end-organ damage that hypertensive patients frequently experience, such as hypertensive retinopathy, cardiac hypertrophy, hypertensive nephropathy, and cerebrovascular problems [105]. Thus, a clinically useful antihypertensive drug that can both safely decrease blood pressure and preserve end organs is still required [105]. Due to its cardioprotective, nephroprotective [106], neuroprotective [107], and retinoprotective properties [108], RES may contribute to endogenous defence mechanisms. While these findings highlight its potential as a treatment for hypertension, further investigation is necessary [109]. Studies on various hypertensive animal models, such as spontaneously hypertensive rats (SHRs), angiotensin II-infused mice, two-kidney one-clip hypertensive rats, and partially nephrectomized rats, have demonstrated that RES exhibits anti-hypertensive properties at doses between 10 and 320 mg/kg body weight per day [110]. However, lower doses (2.5 mg/kg/day) did not produce significant changes in blood pressure [111]. Thandapilly *et al*. explored whether RES could enhance the effects of other antihypertensive drugs by administering low doses of RES with or without hydralazine in SHRs. While RES alone, even at 2.5 mg/kg/day, did not alter systolic or diastolic blood pressure [112], the combination with hydralazine led to a significant reduction in both parameters [113]. Despite the significance of these results, hydralazine is not frequently utilized in clinical settings to treat hypertension. Therefore, to apply these findings to people, it will be crucial to both understand the mechanism underlying these intriguing findings and confirm them with anti-hypertensive drugs that are more frequently prescribed, such as beta blockers, ACE inhibitors, angiotensin receptor blockers, Ca^2+^ channel blockers, and thiazide diuretics. It's interesting to note that several studies that did not find that RES decreased blood pressure still showed other beneficial effects on the vasculature [114, 115]. Age-related and obesity-related declines in endothelial function have been demonstrated to be prevented by RES therapy (2400 mg/kg of diet), since these three variables are major risk factors for endothelial dysfunction [116, 117]. Hypertension affects about 25% of people in industrialized countries [116, 117] and is recognized to play a role in the development of both of these CVDs [118]. Based primarily on preclinical studies, the mechanisms through which RES may lower blood pressure include increasing endothelial NO production [119-121], decreasing oxidative damage and vascular inflammation by upregulating SIRT1 expression in endothelial cells [122], and reducing Ca^2+^ influx [123]. Nevertheless, there is conflicting clinical data about RES's effects on blood pressure. Research demonstrating a drop in blood pressure often only detect a drop in diastolic blood pressure (DBP), not systolic blood pressure (SBP) [124]. This might not be a problem, though, as research suggests that SBP poses a greater risk of CVDs than DBP [125]. Furthermore, as Beshay *et al*. pointed out, certain research on individuals with metabolic issues that demonstrate a drop in SBP [124, 126] may have been the result of metabolic improvements; as a result, RES's direct vasodilator impact cannot be verified. Furthermore, research on individuals with hypertension shown that RES may be used in conjunction with angiotensin-converting enzyme (ACE) inhibitors to effectively lower blood pressure without the need for additional anti-hypertensive drugs [124, 127]. Moreover, meta-analyses indicate that RES administration did not affect SBP or DBP. However, three meta-analyses investigating the antihypertensive effects of RES revealed a dose-dependent impact on SBP. This suggests that future randomized controlled trials (RCTs) examining the effects of RES on blood pressure should employ greater dosages of the supplement. It should be highlighted, nonetheless, that RES administration has been associated with increases in blood pressure, including an elevation of heart rate and DBP [123] and a blunting of mean arterial blood pressure decreases after exercise [126]. It has been proposed that RES probably has more significant effects on a hypertensive population, which would account for the compound's lack of effect on blood pressure shown in several studies [124]. Since then, however, an RCT on hypertensive participants given 300 mg/d of RES has not demonstrated any impact on blood pressure [125]. As a result, the clinical data is still conflicting and inconsistent, and the cause of the variability is still unclear.

#### 1.1.7 Antihypertensive Mechanism of RES

**Fig. (8):**
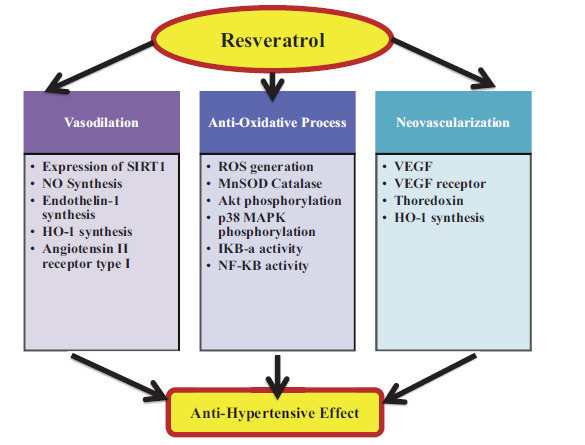
Suggested mechanism for RES's antihypertensive benefits. RES has antihypertensive effects through neovascularization, antioxidation, and vasodilation. Each process's impacted molecular participants are listed. Vascular endothelial growth factor (VEGF), manganese-dependent superoxide dismutase (MnSOD), and hemeoxygenase-1 (HO-1).

A persistently elevated arterial blood pressure is the hallmark of hypertension, a chronic medical illness. In 2000, over 25% of adults worldwide suffered from hypertension [117, 120]. Because it raises the risk of peripheral vascular disease, ischemic heart disease, stroke, and other cardiovascular illnesses, this condition is one of the most important yet avoidable risk factors for early mortality globally. Recent studies suggest that RES may help lower blood pressure through complex mechanisms, including neovascularization, vasodilation, and antioxidant activity [125]. Among its molecular targets, sirtuins play a key role, with SIRT1 being the most extensively studied. RES-induced activation of SIRT1 promotes nitric oxide (NO) production in the vascular endothelium, leading to vasodilation. Beyond its blood pressure-lowering effects, increased endothelial NO also enhances the synthesis of heme oxygenase-1 (HO-1), a precursor of bilirubin. Endothelin-1 (ET-1) is a vasopressor that contributes to vasoconstriction through gene expression changes and synthesis triggered by angiotensin II and mechanical stretch. RES has been shown to counteract these effects by suppressing ET-1 expression, thereby reducing blood pressure [125]. RES inhibits the expression of angiotensin II receptor type 1 in rat vascular smooth muscle cells *via* activating SIRT1. RES's anti-atherogenic qualities and possible lifespan function may be explained by this suppression of the renin-angiotensin pathway [126]. Furthermore, RES supports vascular endothelial function by reducing reactive oxygen species (ROS) [127], activating NF-κB and IκB-α [128], and phosphorylating Akt and p38 MAPK [129]. In ischemic myocardium, RES promotes neovascularization by enhancing the expression of vascular endothelial growth factor and its receptor [130]. Additionally, RES upregulates thioredoxin and HO-1 (Fig. **8**), which work together to strengthen myocardial angiogenesis and antioxidant defense mechanisms [131]. RES's effects on functional vascular endothelial cells suggest that it is a better preventive medication than a therapeutic one when it comes to treating irreversible vascular remodelling. Additionally, RES's quick metabolism guarantees a limited level of bioavailability, underscoring the critical need to find a RES counterpart with higher therapeutic potential for the treatment of hypertension [132].

### 1.2 Calorie Restriction (CR) Improves Cardiac Structure and Function

**Fig. (9):**
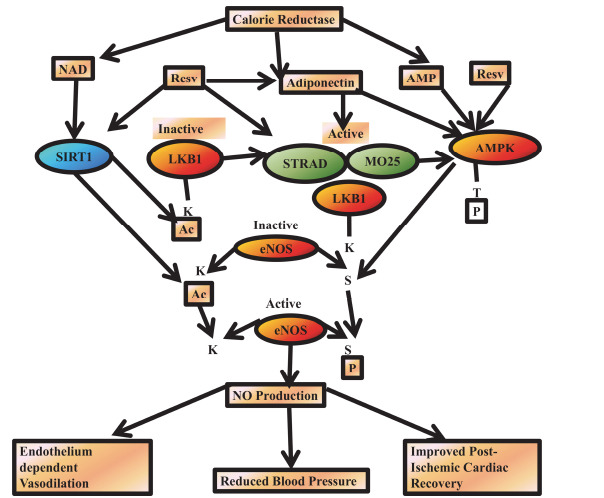
A theoretical theory that connects the physiologic effects of RES and calorie restriction on the cardiovascular system's synthesis of nitric oxide to an integrated molecular signaling network that involves cross-talk between AMPK and SIRT1.

Long-term CR lowers the levels of C-reactive protein, insulin resistance, abdominal obesity, hypertension, hyperlipidemia, and other risk factors for CVD [127, 128, 133]. Furthermore, CR directly improves the cardiovascular system in a variety of ways, with atherosclerosis being one of its main targets. Atherosclerosis, an inflammatory disease of the artery wall, leads to the accumulation of oxidized low-density lipoprotein and wall fibrosis, eventually causing blockages that result in heart attacks and strokes [129]. CR has been demonstrated to not only lower atherosclerosis but also to stop blood pressure from increasing in hereditary and surgical mouse models of hypertension [130-132]. While the exact mechanism underlying this remains unclear, some of the antihypertensive effects of CR may be mediated through an increase in nitric oxide (NO) bioavailability, a powerful vasodilator. In line with this, it has also been demonstrated that calorie restriction enhances NO production in both young and old mice *via* activating endothelial NO synthase (eNOS) [133, 134]. Furthermore, CR improves the vascular function of healthy non-obese individuals [135-137] as well as endothelium-dependent vasodilation in obese patients who already have hypertension [136]. In addition to these specific research initiatives, a wealth of information has been gathered by studying members of the Caloric Restriction Society (CRS), who intentionally restrict their food intake in the hopes of slowing down the aging process [138, 139]. When calories are restricted, the cell's energetic state decreases and its NAD and AMP levels increase (Fig. **9**).

Data from liver cells and tissues [140] indicate that NAD activates SIRT1, which activates LKB1 by removing acetyl groups (Ac) from lysine (K) on LKB1. This improves LKB1's affinity for its STRAD and MO25 subunits, activating LKB1. Because AMPK phosphorylates (P) threonine (T), it becomes more active when AMP levels and LKB1 activity are elevated.

In addition to increasing circulating adiponectin levels, calorie restriction also activates AMPK and LKB1 pathways [141]. In addition to increasing AMPK activity, SIRT1, LKB1, and adiponectin levels, RES also mimics the effects of CR on this signalling network. When AMPK phosphorylates serine (S)1179 and SIRT1 deacetylates K496 and K506 [142], these signalling molecules converge at eNOS, which produces nitric oxide (NO). Benefits of enhanced NO bioavailability on the cardiovascular system include improved post-ischemic recovery of heart function, lowered blood pressure, and endothelium-dependent vasodilation [143].

#### 1.2.1 Antiatherogenic Effects of RES

**Fig. (10):**
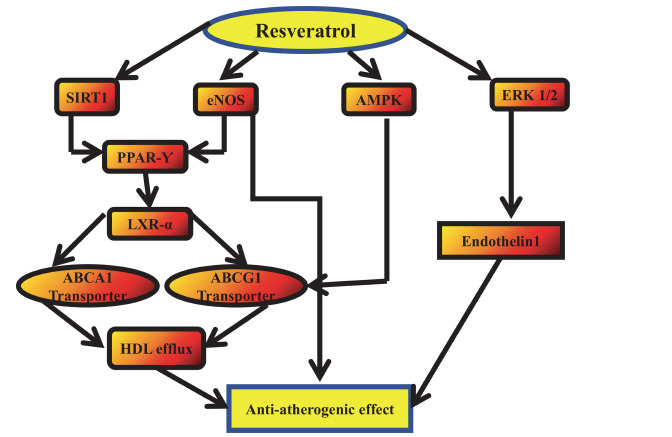
An example of RES's antiatherogenic properties. The serial responses that are first triggered by RES are shown by the black arrows. The antiatherogenic actions of RES are intimately linked to downregulation of the endothelin 1 gene, increased HDL efflux, and eNOS activation. **Abbreviations:** Endothelial nitrous oxide synthase (eNOS); liver X receptors (LXRa); peroxisome proliferator-activated receptor gamma (PPAR-γ); extracellular signal-regulated kinase 1/2 (ERK1/2); sirtuins 1 (SIRT1).

Atherosclerosis, also known as arterial wall inflammation, is a disease brought on by cytokines and mitochondrial signaling that promote endothelial damage in response to redox and hemodynamic stress [143]. All phases of atherosclerosis are mediated by inflammation, and macrophages seem to be important in this condition. The intake of large quantities of lipoproteins causes macrophages to change into foam cells, which is the primary mechanism behind the early development of atherosclerotic lesions [144]. It's interesting to note that RES influences a wide range of chemical substances involved in macrophage lipid metabolism [145]. By transcriptionally regulating COX-2 activity, RES limits inflammation linked to atherosclerosis and, eventually, reduces PGE2 synthesis (Fig. **10**) [146]. PPAR-γ stimulates the maturation of macrophages and the absorption of modified low-density lipoprotein (LDL)15. It also has antiatherogenic effects on smooth muscle cells, endothelial cells, and macrophages [147]. As a result, PPAR-γ agonists could have anti-inflammatory properties that aid in the prevention of atherosclerosis [148].

Remarkably, PPAR-γ is selectively activated by RES. Additionally, LXRs, the main transcriptional regulators of lipid metabolism, are direct transcriptional targets of PPAR-γ and function as sensors of cholesterol [143, 149]. Numerous oxysterols, which are the oxidized derivatives of cholesterol, have been shown in another experimental investigation to act as intermediates in the cholesterol pathway and activate LXR-a [146, 150]. Crucially, RES regulates the process of atherogenesis by amplifying LXR activity. Transmembrane proteins known as ABC transporters assist carry molecules across cell membranes by hydrolyzing ATP and collecting the energy that results [148]. Some ABC transporters, including ABCA1 and ABCG1, aid in cellular cholesterol homeostasis, reverse cholesterol transport, and cholesterol and phospholipid efflux. They also assist prevent the advancement of atherosclerosis in arteries. ABCA1 and ABCG1 are two examples of the lipid transporters that LXRs activate [149]. By regulating the vasoconstrictor endothelin-1 (ET-1), RES also regulates the production of NO, preventing atherogenesis and promoting thromboresistance. Because RES inhibits the extracellular signal-regulated kinase (ERK) 1/2 pathway, it suppresses the synthesis of ET-1, which is a stress-induced protein [150].

#### 1.2.2 Anti-inflammatory Effects of RES

**Fig. (11):**
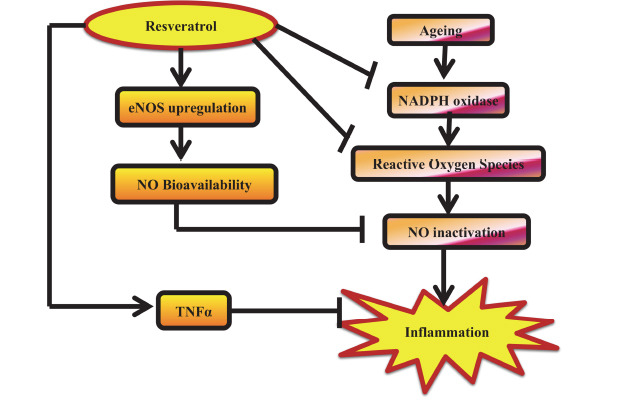
Hypothesized mechanism of RES's anti-inflammatory actions in the aging vasculature. **Abbreviation:** Tumor necrosis factor-alpha (TNF-α).

The significance of age-related changes in addition to the conventional risk factors for the development of CVD is highlighted by recent research [151]. Specifically, low-grade inflammation linked to ageing raises the risk of coronary artery disease and stroke in older individuals [152]. More research confirms the idea that the vascular oxidative stress linked to ageing is caused by elevated NAD(P)H oxidase activity and excess mitochondrial reactive oxygen species (ROS), which also cause endothelial damage and inflammation [153]. NO is a crucial component in age-related alterations and is essential for preserving endothelial cell function. Due to the inactivation of NO by high superoxide concentrations brought on by ageing-induced oxidative stress, there is a significant increase in endothelial cell death, impairment of mitochondrial biogenesis, and vasomotor dysfunction [154]. Endothelium-derived nitrous oxide synthase (eNOS) is expected to protect the cardiovascular system throughout ageing, as mice missing the eNOS gene demonstrate early mortality and premature cardiac ageing (Fig. **11**) [155]. The use of RES supplements has gained popularity recently as a means of mitigating the proatherogenic vascular changes brought on by ageing [156]. RES raises eNOS and improves NO bioavailability, which is consistent with these possible advantages. RES has vasoprotective qualities in mice by restoring the physiological alterations caused by oxidative stress as a result of ageing.

Enhanced HDL efflux, downregulation of the endothelin 1 gene, and eNOS activation are all directly linked to RES's antiatherogenic effects. Vascular NADPH oxidases and the ATP-binding cassette (ABC) transporters A1 and G1 are inhibited by RES [157], and tumor necrosis factor-a expression is downregulated in cardiac and vascular tissues. Additionally, it eliminates the generation of ROS in the vasculature by the mitochondria. ABCA1(G1), NOS, ERK1/2 [158], liver X receptors, PPAR-γ, peroxisome proliferator-activated receptor gamma, SIRT1, sirtuins, and eNOS are all thought to be responsible for these effects [159, 160]. These test results suggest that RES may have anti-inflammatory qualities that help reduce older persons’ chance of dying from atherosclerosis. While RES increases life in yeast and nematodes, this impact is less consistent in other higher order species, according to a meta-analysis that examined the published findings of 19 research on longevity across species [161]. Further study is needed to determine whether RES has any positive effects on human health.

#### 1.2.3 Cardioprotective Effects of RES

Many recent research using animal models of heart disease have revealed that one of the major positive benefits of RES is cardioprotection [162-165]. RES shields cardiomyocytes from oxidative stress, autophagy, apoptosis, and cardiac fibrosis, according to research on animals. In particular, RES limits the generation of ROS by activating SIRT1. Furthermore, RES inhibits oxidative stress in mitochondria and prevents cellular damage by increasing the production of mitochondrial superoxide dismutase (SOD2) [166-169].

The physiological reaction of heart muscle to hemodynamic excess brought on by a variety of physiological and pathological circumstances is known as myocardial hypertrophy. On the other hand, sustained hypertrophy is thought to be a maladaptive process that accelerates work overload and ultimately results in the organism’s mortality [165]. Numerous mechanisms account for the preventive effects of RES against heart hypertrophy. First, small artery remodelling and compliance are reduced by RES [170, 171]. Rats with abdominal aortic bands treated with RES show a regression of pressure overload-induced cardiac hypertrophy and dysfunction [172], which may be because of elevated eNOS/NO expression [173]. Second, when RES prevents oxidative stress from inhibiting liver kinase B1 (LKB1), 50 AMP-activated downstream ignaling molecules are activated more easily. Heart remodeling and needless protein synthesis are prevented by activating AMPK in conjunction with blocking mTOR/70-kDa ribosomal protein S6 kinase signaling [173-175]. Third, RES protects the heart from the development of hypertrophy by influencing the cardiac transcription of the angiotensin II receptor, AT1a [176].

**Fig. (12):**
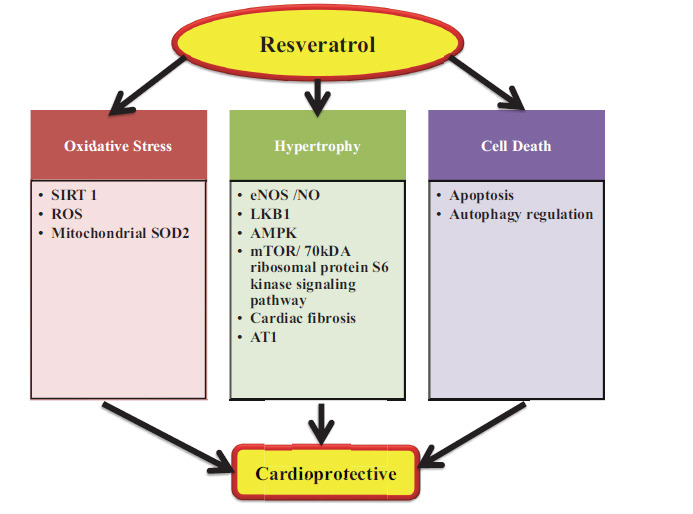
An example of RES’s cardioprotective properties. To achieve its cardioprotective effects, RES reduces autophagy, oxidative stress, hypertrophy, apoptosis, and heart fibrosis. For every process, significant mechanistic actors are outlined. **Abbreviations:** LKB1, liver kinase B1; AT1, angiotensin II receptor 1; AMPK, 50 AMP-activated protein kinase.

There are many circumstances in which RES is shown to have cardioprotective properties. Low ambient temperature is thought to be a significant risk factor for CVD as it causes cardiac hypertrophy and impairment of function [177]. Treatment with RES successfully inhibits these changes by preventing cardiomyocytes from dying. Patients with diabetes frequently have autophagic malfunction; RES, which directly triggers autophagy, thereby benefiting diabetic cardiomyopathy [174-178]. Through the inhibition of the ROS/ERK/TGF-b/periostin pathway, RES lessens myocardial fibrosis in diabetic mice. Furthermore, by upregulating microRNA-130a [179], blocking the upregulation of miR-34a and the miR-34a/Sirt1 pathway during hypoxia/reoxygenation injury [180], lowering oxidative stress, and reducing cardiac inflammation and fibrosis, RES guards against myocardial injuries brought on by myocardial infarction or hypoxia/reoxygenation injury (Fig. **12**).

More recent research has examined whether RES shields the myocardium from exogenous factors like endotoxin lipopolysaccharides or therapeutic drugs. RES protects against cardiac toxicities brought on by doxorubicin or arsenic trioxide [181-183]. Furthermore, RES increases doxorubicin’s cellular absorption, which amplifies its anticancer action [182]. These findings imply that adding RES to doxorubicin treatment might help avoid cardiotoxicity and have a synergistic effect on cancer cells. In conclusion, RES prevents oxidative stress, suppresses autophagy, lowers apoptosis, and improves cardiac fibrosis to safeguard cardiomyocytes. Moreover, RES has a positive effect in preventing heart hypertrophy. Nevertheless, despite positive outcomes in animal models, there are currently few human participants in clinical studies that corroborate RES’s favourable benefits [184-188]. Therefore, more investigation is necessary to understand why the results from experimental animals and people differ. Table **1** provides a detailed evaluation of the possible advantages and limitations of resveratrol in managing cardiovascular diseases. The “Pros” section outlines its therapeutic advantages, including antioxidant activity and cardioprotective properties, supported by current scientific evidence. Conversely, the “Cons” section details its limitations, such as low bioavailability, possible side effects, drug interactions, and a lack of long-term safety data. Each point is accompanied by references to provide evidence-based insights, offering a balanced perspective on resveratrol’s potential in cardiovascular health management [189, 190].

## CONCLUSION

Resveratrol has gained significant attention for its potential benefits in cardiovascular disease management, largely due to its antioxidant, anti-inflammatory, and heart-protective properties. Research indicates that it helps mitigate oxidative stress and inflammation, both of which play crucial roles in the progression of atherosclerosis and other heart-related conditions. Additionally, resveratrol supports vascular health by enhancing endothelial function and boosting nitric oxide availability, which may contribute to better blood pressure regulation. Findings from animal studies further suggest that it could protect the heart muscle and enhance left ventricular function in heart failure models. Despite these promising findings, evidence from human clinical trials remains inconsistent, often constrained by resveratrol's poor bioavailability, stemming from rapid metabolism and limited systemic absorption. While short-term studies have reported modest benefits, such as improvements in blood pressure, lipid profiles, and arterial stiffness, the long-term effectiveness and safety of resveratrol are not well established. Additionally, inconsistencies in dosages, formulations, and study populations complicate the interpretation of outcomes. Concerns about potential drug interactions, particularly with anticoagulants and statins, further limit its applicability in clinical settings. Therefore, while resveratrol holds significant therapeutic promise, rigorous and standardized clinical trials are needed to confirm its efficacy and address existing gaps in evidence.

Recent discoveries highlight the potential of caloric restriction (CR) and resveratrol (RES) in managing high blood pressure, left ventricular hypertrophy (LVH), myocardial ischemia, and metabolic disorders, though more clinical data is needed. Further investigation is essential. RES has demonstrated benefits such as reducing myocardial hypertrophy and fibrosis, improving heart failure symptoms, and enhancing left ventricular function. Additionally, RES has been shown to lower oxidative stress and influence key intracellular signalling pathways, including p38-MAPK, ERK1/2, MKP-1, COX-2, iNOS, Akt-1, and GSK-3β, which may contribute to its cardioprotective effects.RES exhibits various protective properties against CVDs, including anti-inflammatory, antihypertensive, antiatherogenic, and metabolic regulatory effects. Ongoing research could further establish its role in preventing and treating CVDs, as clinical trials have already indicated potential benefits. To confirm its therapeutic value, large-scale clinical studies are needed to determine whether RES supplementation can effectively support cardiovascular health. Ultimately, RES remains a promising candidate for CVD treatment, and further laboratory and clinical investigations will help clarify its full potential. If future clinical trials yield positive results, RES may become an increasingly popular option for both the prevention and treatment of cardiovascular diseases.

## Figures and Tables

**Table 1:** A comprehensive overview of the pros and cons of resveratrol for cardiovascular disease treatment.

**S. No.**	**Concern**	**Description**	**References**
**Pros**			
1	Antioxidant Properties	Resveratrol exhibits antioxidant effects, reducing oxidative stress, which is a key factor in the development of CVD.	[188]
		Resveratrol reduces oxidative stress by neutralizing free radicals, preventing damage to endothelial cells and lipids.	[189]
2	Anti-inflammatory Effects	It possesses anti-inflammatory properties that help mitigate vascular inflammation, contributing to improved cardiovascular health.	[115]
		Suppresses inflammatory markers like NF-κB, CRP, and TNF-α, reducing chronic inflammation associated with CVD.	[172]
3	Blood Pressure Reduction	Resveratrol supplementation has been associated with reductions in systolic blood pressure, particularly in individuals with type 2 diabetes.	[173]
4	Improves Endothelial Function	Enhances nitric oxide (NO) bioavailability, promoting vasodilation and reducing arterial stiffness.	[7]
5	Improvement in Lipid Profile	Studies suggest that resveratrol can positively influence lipid profiles by lowering LDL cholesterol and increasing HDL cholesterol levels.	[8]
6	Enhancement of Endothelial Function	Resveratrol may improve endothelial function, which is crucial for vascular health and the prevention of atherosclerosis.	[174]
7	Potential in Heart Failure Management	Emerging evidence indicates that resveratrol could be beneficial in managing heart failure by improving cardiac function and reducing myocardial stress.	[7]
8	Anti-Platelet Aggregation	Inhibits platelet aggregation, reducing the risk of thrombus formation and associated ischemic events.	[175]
9	Lipid Regulation	Reduces LDL cholesterol oxidation, enhances HDL levels, and decreases triglycerides, contributing to better lipid profiles.	[1]
10	Cardiomyocyte Protection	Protects heart muscle cells from ischemia-reperfusion injury, reducing infarct size and improving recovery.	[176]
11	Anti-Atherogenic Properties	Reduces foam cell formation and inhibits vascular smooth muscle cell proliferation, preventing plaque buildup in arteries.	[177]
12	Anti-Apoptotic Effects	Prevents apoptosis of endothelial cells and cardiomyocytes under stress conditions, preserving cardiac and vascular integrity.	[178]
13	Improves Insulin Sensitivity	Reduces insulin resistance, a risk factor for CVD, by modulating pathways like AMPK and SIRT1.	[179]
14	Reduces Hypertension	Lowers blood pressure by enhancing vascular relaxation and reducing angiotensin II effects.	[180]
15	Prevents Cardiac Hypertrophy	Modulates signalling pathways like mTOR and NFAT to prevent pathological cardiac enlargement.	[181]
16	Reduces Myocardial Fibrosis	Inhibits collagen deposition and fibroblast activity in the myocardium, improving diastolic function.	[182]
17	Improves Mitochondrial Function	Enhances mitochondrial biogenesis and efficiency, reducing energy deficits in the heart.	[183]
18	Enhances Autophagy	Activates autophagy, promoting the removal of damaged cellular components in cardiomyocytes and endothelial cells.	[184]
19	Anti-arrhythmic Effects	Stabilizes cardiac electrical activity, reducing the risk of arrhythmias during stress conditions.	[185]
20	Modulates Gut Microbiota	Promotes a favorable gut microbiota composition, which influences systemic inflammation and lipid metabolism.	[5, 29]
21	Neuroprotective Effects	Reduces risk of stroke by enhancing neurovascular coupling and reducing brain oxidative stress.	[106]
22	Modulation of Gut Microbiota	Resveratrol has been shown to positively affect gut microbiota composition, which plays a role in cardiovascular health.	[186]
23	Improvement in Glycemic Control	Supplementation with resveratrol has been linked to better glycemic control, which is beneficial for cardiovascular health, especially in diabetic patients.	[187]
24	Reduction in C-Reactive Protein Levels	Resveratrol intake has been associated with decreased levels of C-reactive protein, a marker of inflammation linked to cardiovascular risk.	[188]
25	Potential Neuroprotective Effects	Resveratrol may offer neuroprotective benefits that indirectly support cardiovascular health by reducing the risk of stroke.	[189]
**Cons**			
26	Poor Bioavailability	Resveratrol is rapidly metabolized, leading to low bioavailability, which limits its therapeutic effectiveness.	[150, 179]
27	Gastrointestinal Issues	High doses (≥1,000 mg/day) can cause stomach upset, diarrhea, and nausea.	[114]
28	Drug Interactions	Resveratrol may inhibit cytochrome P450 enzymes, affecting the metabolism of various drugs, including statins and anticoagulants.	[190]
29	Pro-Oxidant Effects	Under certain conditions, resveratrol may exhibit pro-oxidant properties, leading to oxidative stress.	[27]
30	Bleeding Risk	Resveratrol might slow blood clotting, increasing the risk of bleeding, especially in individuals with bleeding disorders or those undergoing surgery.	[191]
31	Insufficient Long-term Safety Data	Long-term effects of resveratrol supplementation are not well-studied, raising concerns about its chronic use.	[192]

## LIST OF ABBREVIATIONS

ABC: ATP-binding Cassette
ABCA1: ATP-binding Cassette Transporter A1
ACE: Angiotensin Converting Enzyme
ADMA: Asymmetric Dimethylarginine
AF: Atrial Fibrillation
AhR: Aryl Hydrocarbon receptor
Akt-1: *Alpha Serine/Threonine-protein Kinase*
AMP/ATP: Adenosine Monophosphate, Diphosphate, and Triphosphate
AMPK: Adenosine Monophosphate-activated Protein Kinase
AMPK-NO: Adenosine Monophosphate-activated Protein Kinase Nitric Oxide
Ang II: Angiotensin
ApoB: Apolipoprotein B-100
ARE: Antioxidant Response Element
AT1: Angiotensin II Receptor 1
BAEC: Bovine Aortic Endothelial Cells
BH4: Tetrahydrobiopterin
BMI: Body Mass Index
BNP: B-type Natriuretic Peptide
BNP-45: B-type Natriuretic Peptide-45 Levels
BP: Blood Pressure
BW: Body Weight
C: Control Group
CAD: Coronary Artery Disease
CamKK2: Calcium/Calmodulin-dependent Kinase 2
cAMP: Cyclic Adenosine Monophosphate
CF: Calcium Fructoborate
CK: Creatine Kinase
COX: Cyclooxygenase
CR: Calorie Restriction
CRP: C-reactive Protein
CRS: Caloric Restriction Society
CVDs: Cardiovascular Diseases
CYP: Cytochromes P450
CYP1A1: Cytochrome P450 1A1
CYP7A1: Cholesterol 7α-hydroxylase
DBP: Diastolic Blood Pressure
DDAH: Dimethylarginine Dimethyl Amino Hydrolase
DDSs: Drug Delivery Systems
DNA: Deoxyribonucleic Acid
eEF2: Eukaryotic Elongation Factor-2
EF: Ejection Fraction
eNOS: Endothelial Nitric Oxide Synthase
ERK: Extracellular Signal-regulated Kinase
ET: Endothelin
FKN: Fractalkine
FMD: Flow Mediated Dilation
FOX: Forkhead
GE: Grape Extract
GLUT-4: Glucose Transporter
GSK-3β: Glycogen Synthase Kinase-3 Beta
GSK: Glycogen Synthase Kinase
HCAECs: Human Coronary Arterial Endothelial Cells
HDL-C: High-density Lipoproteins-cholesterol
HF: Heart Failure
HF-PEF: HF with Preserved Ejection Fraction
HFD: High-fat Diet
HIF1α: Hypoxia-inducible Factor 1-alpha
HMG-CoA reductase: 3-hydroxy-3-methyl-glutaryl-CoAreductase
HNE- 4: Hydroxy-2-Nonenal
HO-1: Haem Oxygenase 1
HUVECs: Human Umbilical Vein Endothelial Cells
ICAM: Intercellular Adhesion Molecule
IFN-γ: Plasma Interferon
IL: Interleukin
IL-1β: Interleukin 1 beta
iNOS: Inducible NOS
IP3: Inositol Triphosphate
IR: Insulin Resistance
KLF2: Krüppel-like Factor 2
LDH: Lactate Dehydrogenase
LDL-C: Low-density Lipoproteins-cholesterol
LDL-R: Low Density Lipoproteins Receptors
LDLs: Low Density Lipoproteins
LKB1: Kinase Liver Kinase B1
LVEF: Left Ventricle Ejection Fraction
LVH: Left Ventricular Hypertrophy
LXRs: Liver X Receptors
M1: Monocytes
M2: Macrophages
MAP: Mitogen-activated Protein
MAPK: Mitogen-activated Protein Kinase
MCP-1: Monocyte Chemo-attractant Protein-1
MEFs: Mouse Embryonic Fibroblasts
MI: Myocardial Infarction
miRNAs: microRNAs
MKP-1: Kinase Phosphatase-1
MLC: Myosin Light Chain
MMP-9: Matrix Metalloproteinase-9
MnSOD: Manganese-dependent Superoxide Dismutase
mTOR: Mammalian Target of Rapamycin
MYPT1: Myosin Phosphatase-targeting Subunit 1
NAFLD: Non-alcoholic Fatty Liver Disease
NF-κB: Nuclear Factor-kappaB
NFAT: Nuclear Factor of Activated T-cell
NIH: National Institute of Health
NO: Nitric Oxide
NOS: Nitric Oxide Synthase
NQO1: Haem Oxygenase 1
NQO1: Quinone Oxidoreductase 1
NRF2: Nuclear Factor-E2-related Factor-2
NT: Nitrotyrosine
NTS: Nucleus Tractus Solitarii
p38- MAPK: Mitogen-activated Protein Kinase
p70S6K: p70S6 Kinase
PAI-1: Plasminogen Activator Inhibitor Type 1
PBMCs: Peripheral Blood Mononuclear Cells
PGC-1α: Peroxisome Proliferator-activated Receptor-γ Co-activator 1α
PGE2: Prostaglandin E2
PGP: P-glycoprotein
PKC: Protein Kinase C
PPAR-γ: Peroxisome Proliferator-activated Receptor Gamma
RAAS: Renin-Angiotensin Aldosterone System
RAS: Renin-Angiotensin System
RCT: Randomized Clinical Trials
RES: Resveratrol
ROS: Reactive Oxygen Species
SBP: Systolic Blood Pressure
SDS: Polyacrylamide Gel Electrophoresis
SEM: Scanning Electron Microscopy
SERCA: Sarcoplasmic/Endoplasmic Reticulum Ca-ATPase
SHR: Spontaneously Hypertensive Rat
SIRT: Silent Information Regulator of Transcription
SMCs: Smooth Muscle Cells
SOD: Superoxide Dismutase
STZ: Streptozotocin
T-proBNP: N-terminal Prohormone of Brain Natriuretic Peptide
TAC: Transverse Aortic Constriction
TBST: Tris-buffered Saline Tween
TL: Tibia Length
TMAO: Trimethylamine-n-Oxide
TNF: Tumor Necrosis Factor
UCP2: Uncoupling Protein 2
UGT: Uridine Diphosphate Glucuronyosyl Transferase
VCAM-1: Vascular Cell Adhesion Molecule-1
VEGF: Vascular Endothelial Growth Factor
VSMC: Vascular Smooth Muscle Cell
WHO: World Health Organization
WV: Ventricular Weight
ZDF: Zucker Diabetic Fatty
